# Metabolic engineering for recombinant major ampullate spidroin 2 (MaSp2) synthesis in *Escherichia coli*

**DOI:** 10.1038/s41598-017-11845-2

**Published:** 2017-09-12

**Authors:** Hao Cao, Shafaq Parveen, Ding Ding, Haijun Xu, Tianwei Tan, Luo Liu

**Affiliations:** 1 Beijing Bioprocess Key Laboratory, Beijing University of Chemical Technology, Beijing, 100029 P. R. China; 2 Institute of Food Science and Technology, Chinese Academy of Agricultural Sciences, Beijing, 100193 P. R. China

## Abstract

In this research, metabolic engineering was employed to synthesize the artificial major ampullate spidroin 2 (MaSp2) in the engineered *Escherichia coli*. An iterative seamless splicing strategy was used to assemble the MaSp2 gene, which could reach 10000 base pairs, and more than 100 kDa protein was expected. However, only 55 kDa recombinant MaSp2 was obtained. Because MaSp2 is rich in alanine and glycine residues, Glycyl/alanyl-tRNA pool and extra amino acids adding were adopted in order to supplement alanine and glycine in the protein translation process. With the supplementary alanine and glycine (0.05 wt%) in the medium, MaSp2 constructed in pET28a(+) and Gly/Ala-tRNA constructed in pET22b(+) were co-expressed in *Escherichia coli* BL21 (DE3). As results, the artificial MaSp2 with 110 kDa molecular weight was obtained in the present work. This work demonstrates a successful example of applying metabolic engineering approaches and provided a potential way with the enhanced Glycyl/alanyl-tRNA pool to achieve the expression of high molecular weight protein with the repeated motifs in the engineered *Escherichia coli*.

## Introduction

Spider silk is a kind of biomaterial with unique mechanical properties. Spider silk shows outstanding strength, toughness, elasticity, such as it is five times stronger than steel and three times tougher than the Kevlar fiber by same weight^1^. Besides, spider silk is a suitable candidate in medical applications due to its biocompatibility and biodegradability^2^. Spider silk can be used to make parachute cords, cable block, and body armor in military, to make sutures for wounds, vessel for drug delivery, and scaffolds for tissues in medicine^3^.

Up to now, because of the desired mechanical properties and the known protein sequence, the major ampullate spidroin (MaSp) attracts more attention than other spidroins. The sequence of MaSp is highly modular with long repetitive sequence, which includes poly-alanine (A_n_), poly-glycine/alanine ((GA)_n_), GPGXX(X = G, Q, Y, A, S), and GGX (X = L, Y, S, A)^4–6^. MaSp includes two main proteins: major ampullate spidroin 1 (MaSp1) and major ampullate spidroin 2 (MaSp2)^7^. The differences between MaSp1 and MaSp2 are the proline content in sequence and polymeric pattern. MaSp1 contains few proline residues (<1%) and shows high strength, whereas MaSp2 is a proline-rich protein (~9%) with high elasticity, because the characteristic amino acid sequence of MaSp2 is GPGXX, which forms the β-turn spiral structure to enhance the elasticity of dragline fiber^8–11^.

Unfortunately, spider is a kind of territorial and aggressive creature. Therefore, it is difficult to produce spidroin through farming spider like silkworm^12, 13^. Thus, some researchers began to focus on the synthesis of spidroin fibers by metabolic engineering strategy. However, from the view of biosynthesis, efficient production of high molecular weight spider silk protein with the repeat motifs is difficult. Most heterologous expression systems are plagued by low expression levels for a variety of reasons, including instability of cloning, translational pausing and depletion of amino acid and/or tRNA pools^14–16^. As a successful example, in 2010, Xia *et al*. expressed 284.9 kDa recombinant MaSp1 of the spider *Nephila clavipes* and spun into the fiber which displayed the improved mechanical properties; but compared with natural silk fiber, the elasticity of artificial MaSp1 was still lack^17^. The elasticity of MaSp is dependent on intervening glycine-rich repeats such as the GGX motifs of MaSp1 and the GPGXX motifs of MaSp2^18^. Liu *et al*. demonstrated that proline-containing GPGXX motifs contribute to the better elasticity of MaSp2 than that of MaSp1^19^. And Rauscher *et al*. also identified proline as the primary determinant of the elastin-like properties of MaSp^20^.

Several genetic codes of MaSp2 were obtained by genetic engineering and recombinant DNA technology from *Latrodectus hesperus, Latrodectus geometricus, Nephila madagascariensis, Nephil a senegalensis* and *Nephila clavipe*s spiders^21–23^. Santos-Pinto *et al*. isolated and purified the MaSp2 with 269 kDa from the native major ampullate silk of *Nephila clavipe*s spider^24^. However, few studies reported the expression of the recombinant MaSp2 in heterologous hosts. In the present work, the tandem repeated MaSp2 gene of the spider *Nephila clavipe*s was assembled, and the recombinant MaSp2 (up to 110 kDa) was expressed in the metabolically engineered *Escherichia coli*, by supplementary of appropriate amino acids and enhanced tRNA pool. The 110 kDa MaSp2 product showed the potential to be spun in fibers, which will be characterized in future analyses. Moreover, heterologous expression could pave the way on the production of spidroin at the industrial scale.

## Results and Discussion

### Design and assemble the gene of MaSp2

In order to construct the large size gene with the repeated gene fragments, the iterative seamless splicing strategy was developed through adding the flanking restriction enzyme sites, which are compatible but not regenerable. This strategy could support the head-to-tail assembly of the target sequences and allow the mixing of any DNA cassette/module at any required ratios. The isocaudamer can produce the conservative sticky end by recognizing the different DNA sequences. As the DNA sequence of MaSp2 was composed by the tandem repeats, the flanking sequence has only minor effects. The monomer sequence of MaSp2 was designed as follows: *Nde*I and *Bfa*I are underlined. *Eco*RI and *Xho*I in italic are used for cloning and assembly.

CTCG***GAATTC***TCATATGGTCCTGGCCAACAAGGTCCATCTGGTCCTGGCTCTGCAGCTGCAGCAGCTGCTGCAGCTGGTCCAGGTGGCTATGGTCCTGGCCAGCAAGGTCCAGGTGGCTAGTAACGACT***CTCGAG***TCGG

*Bfa*I could recognize a specific sequence CTAG and *Nde*I could recognize a specific sequence CATATG, but both of them could produce the same sticky end TA. After ligation, the sequence was CTATG, which was a part of the coding sequence (GGC TAT GGT) and no more digestible by restriction enzymes. Moreover, GGC TAT GGT sequence codes Gly-Tyr-Gly peptide chain, which is the basic peptide unit of MaSp2.

**Figure 1:**
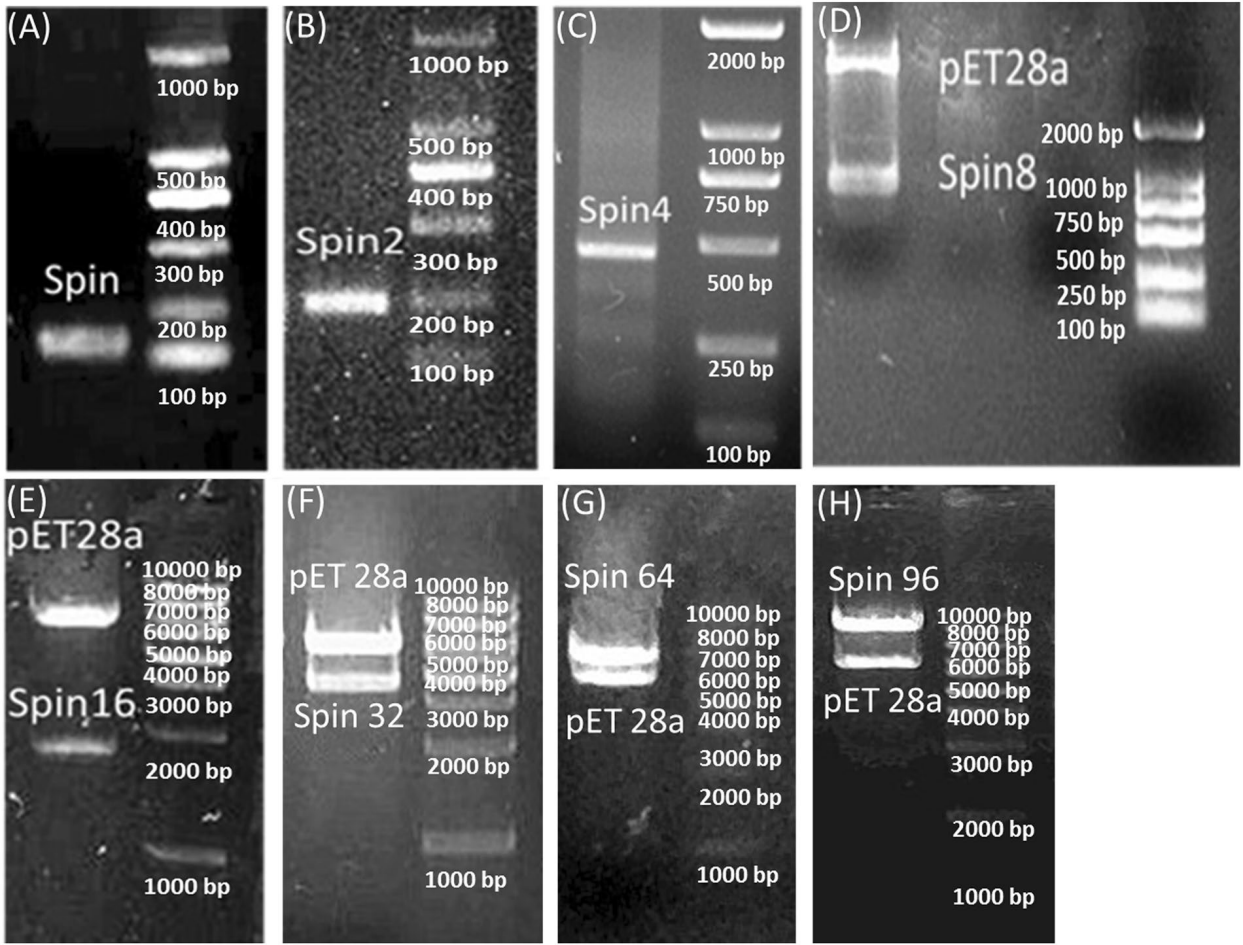
Nucleic acid electrophoresis of the recombinant MaSp2 gene with different tandem spins. MaSp2 gene with Spin (**A**), Spin 2 (**B**), Spin 4 (**C**), Spin 8 (**D**), Spin 16 (**E**), Spin 32 (**F**), Spin 64 (**G**) and Spin 96 (**H**). Each of gels (**A**–**H**) with full-length maker lane was cropped from the different gels. The original gels were presented in Supplementary Figure 1.

Following the same strategy, the gene fragments of MaSp2 with different tandem spins, such as 2, 4, 8, 16, 32, 64 and 96, were constructed. Most tandem times of the gene reached up to 96 spins (Fig. 1).

### Protein expression and product verification

**Figure 2:**
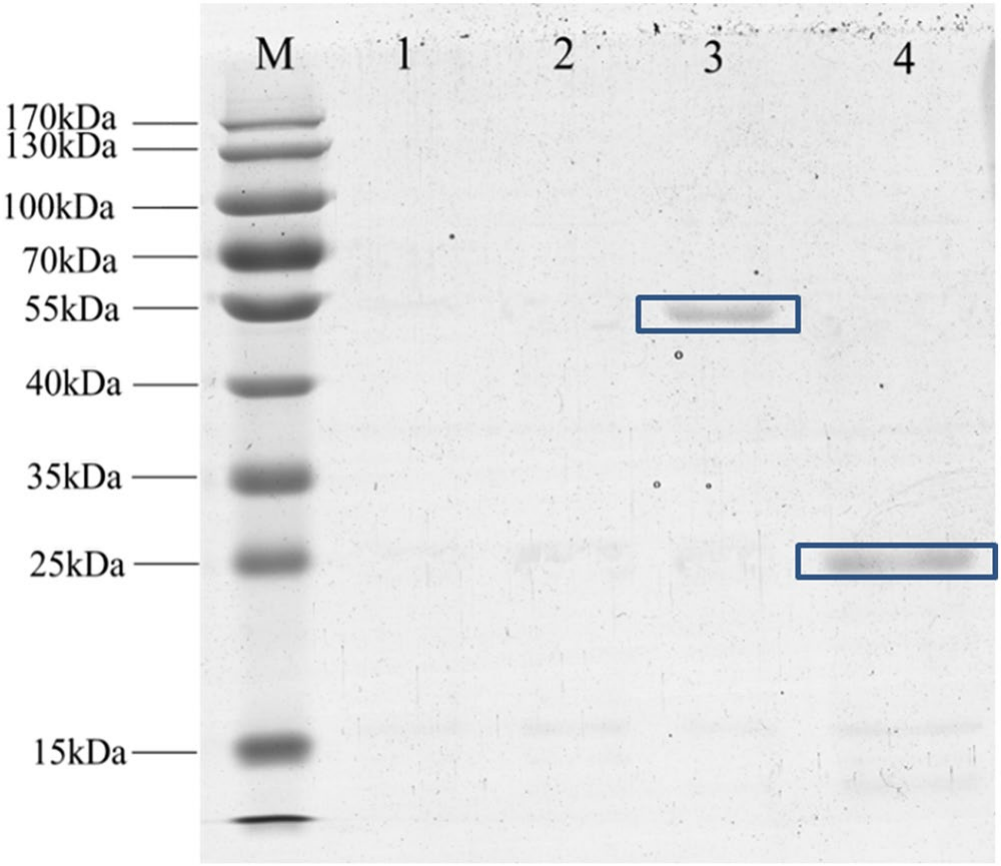
SDS-PAGE analysis of the recombinant MaSp2. Lane (M): Full-length marker; Lane (1): Empty vector. Lane (2): MaSp2 with Spin32. Lane (3): MaSp2 with Spin16. Lane (4): MaSp2 with Spin8. The target proteins were highlight by box.

As shown in Fig. 2, the recombinant MaSp2s in pET28a-Spin8 and pET28a-Spin16 were expressed and the molecular weight is approximately 25 kDa and 55 kDa, respectively. In contrast, the recombinant MaSp2s in pET28a-Spin32, pET28a-Spin64 and pET28a-Spin96 could not be expressed. Moreover, the amino acid composition of both MaSp2 with Spin8 and MaSp2 with Spin16 were close to the theoretical percentage, even though measuring error and the extra amino acid composition (e.g. His-Tag) could affect the results of measured percentage (Table 1).

**Table 1:** Analysis of amino acid composition of MaSp2 with different spins.

Amino acid	Theoretical percentage of MaSp2 (%)	Measured percentage of MaSp2 with Spin8 (%)	Measured percentage of MaSp2 with Spin16 (%)	Measured percentage of MaSp2 with Spin32 (%)
Glycine	37.14	33.48	38.24	38.79
Alanine	22.86	22.67	23.62	19.32
Proline	17.14	16.44	13.55	15.97
Glutamine /Glutamic acid	11.43	14.75	13.59	12.98
Tyrosine	5.71	4.78	4.98	5.30
Serine	5.71	7.87	6.03	7.65

### Effect of supplemental amino acid into the medium on the expression of MaSp2

**Figure 3:**
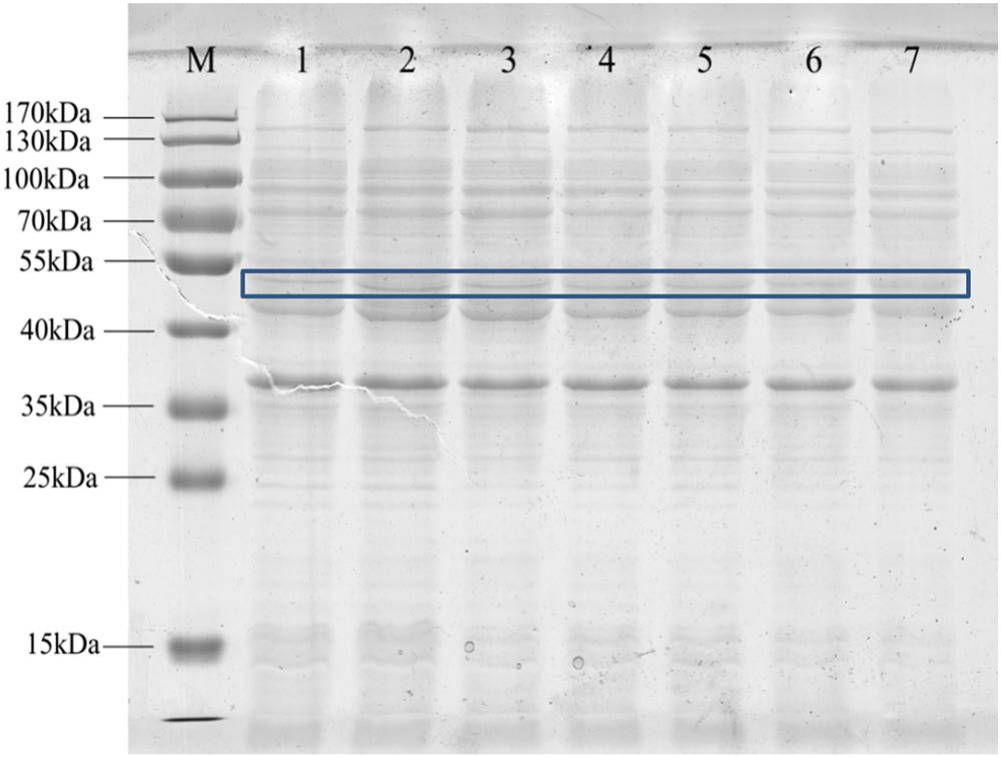
SDS-PAGE analysis the Spin16 protein expression in different amino acid content. Lane (M): Full-length marker; Lane (1): No supply amino acid; Lane (2–7): Supply 0.05 wt%, 0.1 wt%, 0.2 wt%, 0.3 wt%, 0.4 wt%, 0.5 wt% alanine and glycine respectively. The target proteins were highlight by box.

**Figure 4:**
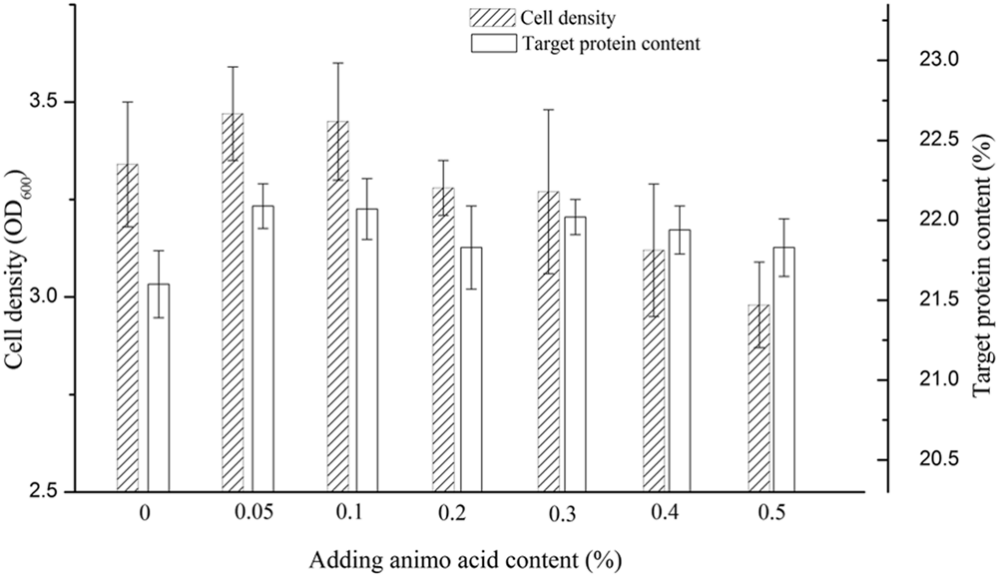
Effects of supplementary amino acids on cell growth and protein expression. The data are shown as average ± standard deviation (n = 3).

In order to investigate the effect of supplemental amino acid on the expression of MaSp2, the different amounts (0.05 wt%, 0.1 wt%, 0.2 wt%, 0.3 wt%, 0.4 wt%, 0.5 wt%) alanine and glycine were added into the medium and then to detect the expression of MaSp2 with Spin16. MaSp2s with Spin16 could be expressed in all of tested amounts of supplemental amino acid (Fig. 3). As shown in Fig. 4, the expression amount of MaSp2 reaches the highest value (22.09%) at 0.05 wt% supplemental amount of amino acid; but with the increasing supplemental amount of alanine and glycine, the expression amount of MaSp2 slightly decreases. Additionally, the cell growth was promoted at 0.05 wt% adding amount of amino acid, but the cell growth was obviously inhibited by excess alanine and glycine (more than 0.2 wt%). However, with 0.05 wt% alanine and glycine adding, MaSp2 with Spin32 still could not be expressed under the same expression conditions with the MaSp2 with Spin16.

**Figure 5:**
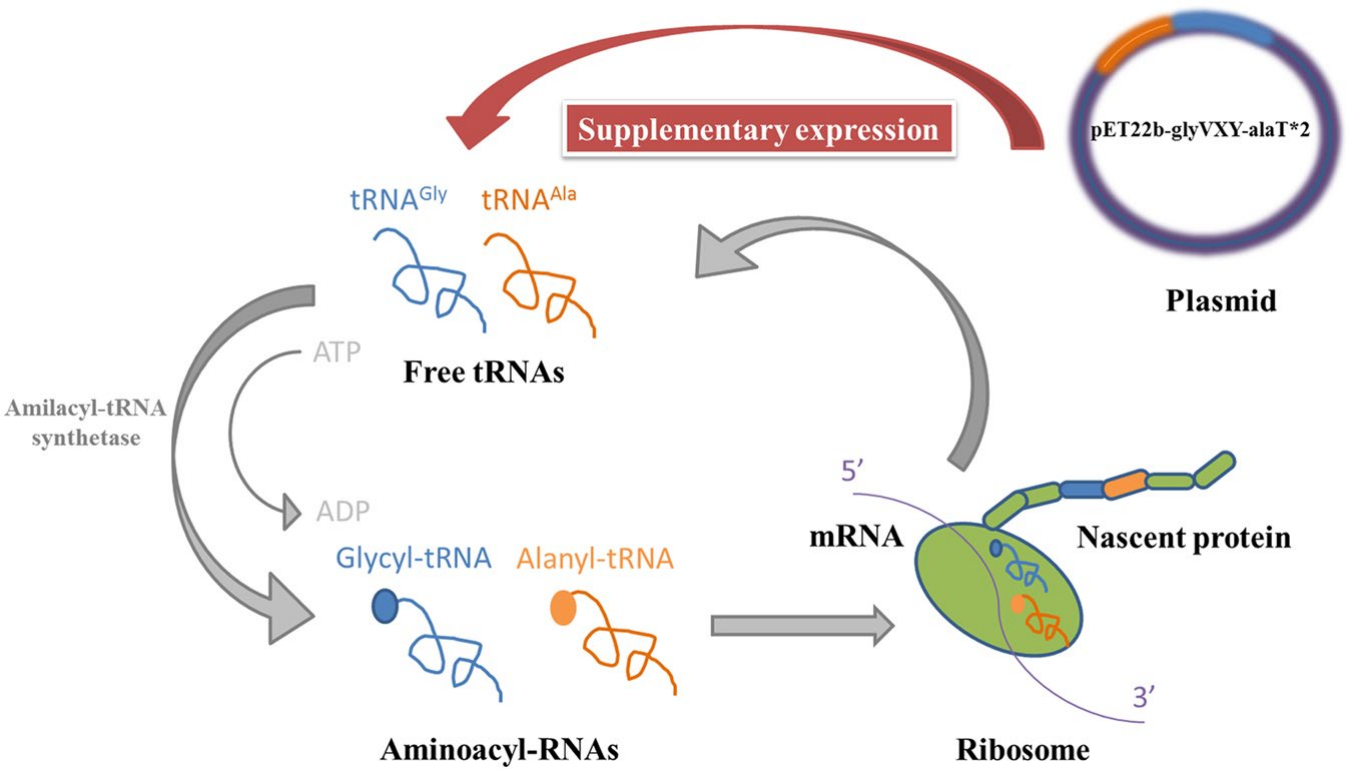
Sketch map of nascent protein synthesis with enhanced tRNA pool.

**Figure 6:**
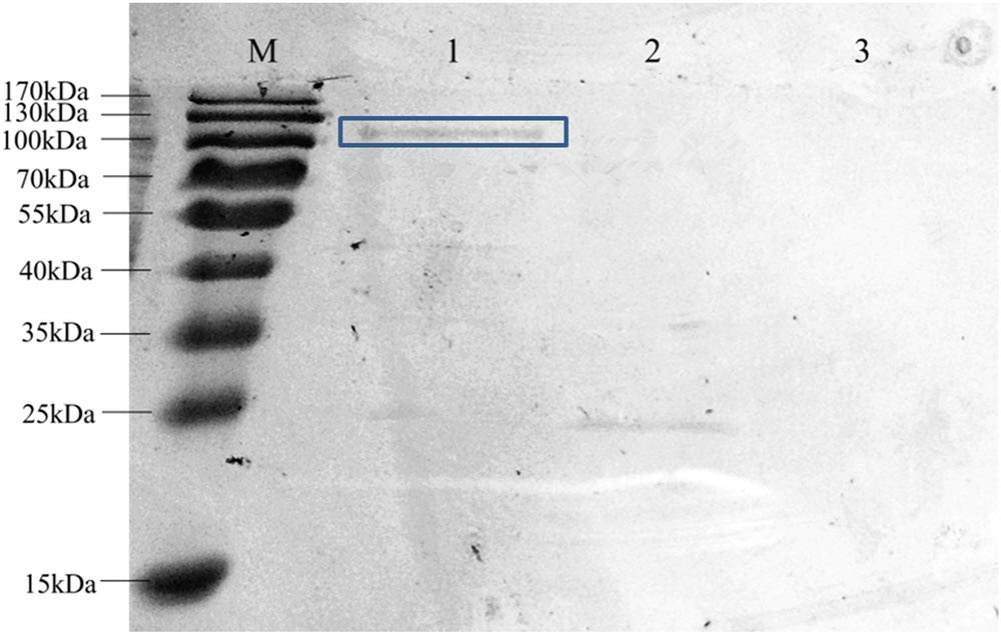
SDS-PAGE analysis of MaSp2 with Spin32. Lane (M): Full-length marker. Lane (1): MaSp2 with Spin32 in the enhanced tRNA system. Lane (2): MaSp2 with Spin32 in the control system. Lane (3): Empty vector. The target protein was highlight by box.

It is not easy to express the high molecular weight heterologous protein in the host. Additionally, as the repeated amino acid sequence, the translation process of MaSp2 might need sufficient tRNAs and amino acids. In the present work, directly adding amino acids did not work well as expected. The possible reason could be that the cell system need tRNAs to transport specific amino acids to express the target protein in the translation process, however, *E. coli* could not provide sufficient Glycyl- and Alanyl- tRNA to express the MaSp2 with high molecular weight and Gly-Ala repeated sequence. In order to supply additional tRNA for protein translation, pET22b-glyVXY-alaT*2 including the code of Glycyl/alanyl-tRNA was transformed into *E. coli* to increase the certain tRNA pool (Fig. 5). Further, pET28a-Spin32 and pET22b-glyVXY-alaT*2 were simultaneously transferred into *E. coli* to co-express the MaSp2 with Spin32 following the previous strategy^25, 26^. As the results, the MaSp2 with Spin32 (110 kDa) was successfully expressed in *E. coli* with enhanced Glycyl/alanyl-tRNA pool and additional supplemental alanine and glycine with 0.05 wt% (Fig. 6). After purification and lyophilization, the expression amount of the MaSp2 with Spin32 was 150 mg/L; and the amino acid composition of the MaSp2 with Spin32 was shown in Table 1.

Metabolic engineering has been widely employed to express the target protein for overcoming the limitation of native host. However, the expression of some especial proteins (e.g. high molecular weight protein) is still challenge towards heterogeneous host. Spidroin, as a high molecular weight protein, has the repeated Gly and Ala amino acid sequences. The main technical challenges of the production of spidroin at the present stage of metabolic engineering are to construct large size genes and expression of high molecular weight protein. In this paper, *E. coli* was metabolically engineered to produce recombinant spider silk protein MaSp2 of up to 110 kDa from *N. clavipes*. The similar size of MaSp2 from *N. clavipes* (110 kDa) was observed only in mammalian cells, previously^16^. The production by using *E*. *coli* as expression host could be more efficient. In the future work, the mechanic properties of the artificial MaSp2 will be analyzed. On the other hand, the MaSp2 with Spin64 and with Spin96 will be further investigated for expression from the view of the heterogeneous host and plasmid. Furthermore, relevant tRNA supplementary expression strategy, which enrich the relevant Aminoacyl-tRNA in the cell in order to synthesize nascent protein, could be employed into the expression of protein with the excess amino acid composition.

## Methods

### Strains, plasmids, and chemicals

*E. coli* strain W3110 was preserved in our laboratory for isolation of genomic DNA. *E. coli* strain BL21 (DE3) and Top10 were obtained from Tiangen Ltd. (China), for protein expression, or plasmids amplification, respectively. Expression vector pET28a(+) and pET22b(+) were purchased from Novagen (Germany).

All restriction enzymes, pyrobest DNA polymerase were purchased from Takara, and T4 DNA ligase was purchased from New England Biolabs (USA). Plasmid miniprep kits, PCR purification kits and gel extraction kits were purchased from Omega bio-tek (USA). The oligonucleotide primers for polymerase chain reaction (PCR) were synthesized by Biomed Ltd. (China).

### Plasmids construction

**Figure 7:**
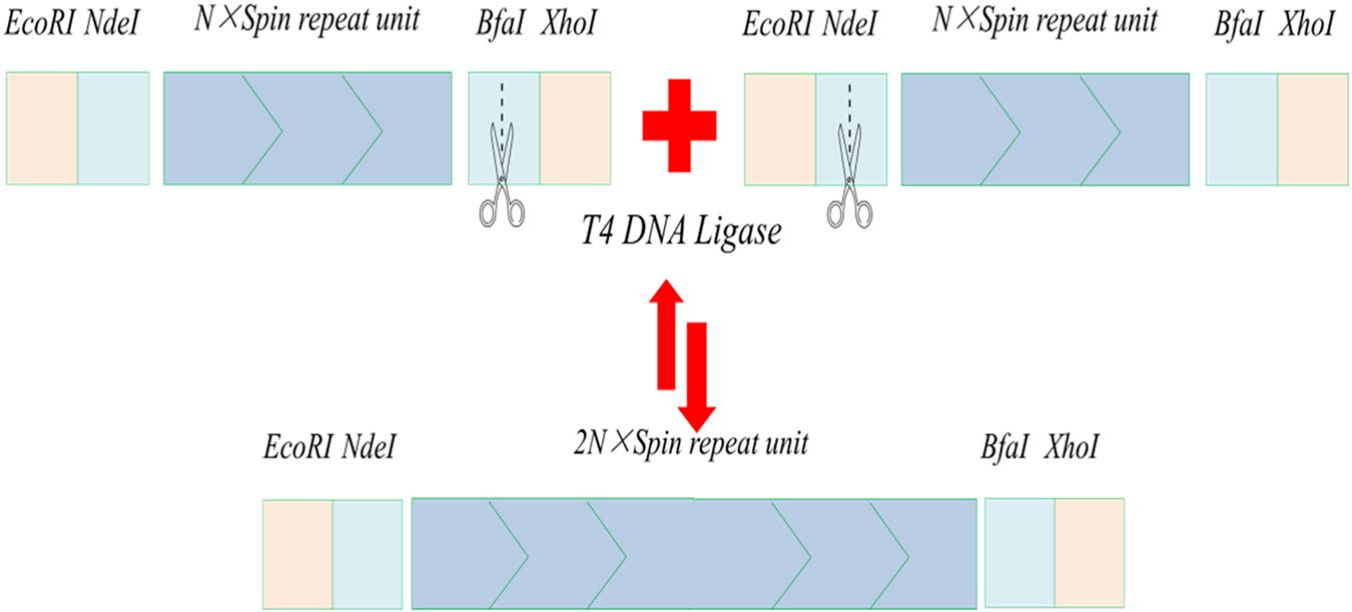
Sketch map of gene splicing using isocaudamer.

As spider protein has a high molecular weight and includes repeated amino acid sequence, head-to-tail strategy was used to construct plasmid with repeated oligonucleotide. The sequence of MaSp2 of *Nephila clavipe* was obtained from NCBI (GenBank accession no. P46804). The synthesis of monomeric gene spin was done by overlaps PCR, and the used primers are listed in Table 2. The PCR product monomer was digested with *Eco*RI and *Xho*I, and cloned into pET28a(+). In order to construct the large coding units with many repeats, the monomer spin after sequencing was subjected to the “head-to-tail” strategy, employing two compatible, but non-regenerable restriction enzyme sites (*Nde*I and *Bfa*I), i.e. isocaudamer. The plasmid containing monomer was digested with *Eco*RI and *Xho*I to release the gene insert. The insert was separated into the equal portion, and digested by *Nde*I or *Bfa*I, respectively. After ligation, the product was cloned into plasmid pET28a(+) (previously digested with *Eco*RI and *Xho*I). This strategy was used to build large synthetic spider silk-like tandem repeat sequences from small double-stranded monomer (oligomer) DNAs flanked by compatible, but nonregenerable restriction sites. By doing so, the sequences of the artificial MaSp2 with 2, 4, 8, 16, 32, 64 and 96 repeats were constructed. The recombinant plasmids containing the different silk-like insert fragments were subjected to restriction digestion with *Eco*RI and *Xho*I to release the gene insert. The released products were separated and characterized by agarose gel. Schematic diagram of the splicing process is shown in Fig. 7.

**Table 2:** Primers used for synthesis monomeric gene Spin.

Oligonucleotide	Sequence (5′–3′)
LZS1-F	CTCGGAATTCTCATATGGTCCTGGCCAAC
LZS1-R	TGCAGAGCCAGGACCAGATGGACCTTGTTGGCCAGGACCATATGAGAA
LZS2-F	CATCTGGTCCTGGCTCTGCAGCTGCAGCAGCTGCTGCAGCTGGTCCAGGTGGCTATG
LZS2-R	CTAGCCACCTGGACCTTGCTGGCCAGGACCATAGCCACCTGGACCAGC
LZS3-R	CCGACTCGAGAGTCGTTACTAGCCACCTGGACCTTGC
glyVXY-HindIII-F	CCCAAGCTTTAACGACGCAGAAATGCGAA
glyVXY- NotI-R	ATAGTTTAGCGGCCGCTAAGATTACAGCCTGAGGCTGTG
alaT-SalI-F1	ACGCGTCGACGGTCGGTGGTTCAAGTCCAC
alaT-XhoI-R1	CCGCTCGAGGTTCAGTGTTTCAATTTTCA
alaT-NotI-F2	ATAAGAATGCGGCCGCCGCACCCCTGATAAGGGTGA
alaT-XhoI-R2	CCGCTCGAGACTCACGAACAACTTTCGTT

The *glyVXY* genes were amplified from the genomic DNA of *E. coli* W3110 using the primers glyVXY-HindIII-F and glyVXY- NotI-R. The amplified DNA, after digestion with *Hind*III and *Not*I, was cloned into plasmid pET22b(+) to construct pET22b-glyVXY.

The *alaT1* and *alaT2* genes were amplified from the genomic DNA of *E. coli* W3110 using the primers alaT-SalI-F1, alaT-XhoI-R1 and alaT-NotI-F2, alaT-XhoI-R2. The amplified DNA alaT1, alaT2 were digested with *Sal*I and *Xho*I separately, then ligated together to construct the alaT*2 gene, and amplified with the primers alaT-*Sal*I-F1 and alaT-*Xho*I-R2. The amplified alaT*2 was digested with *Xho*I and *Not*I, to cloned into plasmid pET22b-glyVXY which had been double digested with *Xho*I and *Not*I and been purified by agarose gel. Thus, we obtained plasmid pET22b-glyVXY-alaT*2.

### Transformation and expression of plasmids

The pET28a(+) with the DNA fragment of the recombinant MaSp2 was transformed into *E. coli* BL21 (DE3) by heat shock (42 °C 90 s). In the co-expression case, the *E. coli* cells including the pET28a(+) with the DNA fragment of the recombinant MaSp2 were washed by 10% glycerol and the plasmid pET22b-glyVXY-alaT*2 was transformed into the *E. coli* cells by electro-transformation at 2.5 kV.

### Protein expression

*E. coli* cells were grown in the 250 mL flask containing 50 mL of Luria Broth (LB) medium at 30 °C placed in an incubator shaking at 170 rpm. When the OD_600_ reached 0.6, cells were induced with 1 mM IPTG. After induction at 30 °C for 6 h with shaking at 170 rpm, bacteria solution was centrifuged (8000 rpm) for 10 min. The sediments were taken and were suspended by phosphate buffer saline (PBS) (pH 8.0). Cells were lysed by sonication (3s-3s-70 cycles). After sonification, sample was centrifuged (10000 rpm) for 10 min and the supernatant was collected for the analysis of protein content based on the optical density method.

With increasing number of the repeated units of MaSp2, the demand for alanine and glycine further increased. In order to facilitate the protein translation process, the different amounts (0.05 wt%, 0.1 wt%, 0.2 wt%, 0.3 wt%, 0.4 wt%, 0.5 wt%) alanine and glycine were added into the medium after inducing with IPTG.

### Protein purification

Each sample was loaded on a 25 mL chromatography column containing 2 mL Ni-NTA His Bind resin. The proteins were eluted using an imidazole step gradient. Low concentrations of imidazole binding buffer around 10 mM–30 mM was used to remove impurities in the successive washes. While the higher concentrations about 300 mM of imidazole was used to elute the recombinant proteins. The resin was stripped of the nickel ions using 100 mM EDTA and the regeneration of column was performed by using 500 mM NiSO_4_.

### Amino Acid analysis

1 mL concentrated hydrochloric acid was added in 1 mL desalination protein sample solution and treated at 155 °C, for 22 h. The hydrolysate was centrifuged, and 100 μL supernatant was taken to dry, then it was dissolved in 200 μL acetonitrile water solution in the ratio of (75:25) and blended by a vortex. After centrifugation at 12000 rpm for 5 min the supernatant was analyzed by Liquid Chromatogram(LC)-mass spectrum (MS). LC (LC-20AD, Shimadzu, Japan) coupling to MS (5500 Q TRAP LC-MS/MS, Allen-Bradley, America) was performed using a binary gradient solvent system of Water (0.1% formic acid) and acetonitrile (2.5 mmol/L ammonium formate and 0.1% formic acid). The detailed gradient description was shown in Table 3. Separation was performed using BEH Amide 1.7 μM 100 × 2.1 mm column and the column temperature was 50 °C.

**Table 3:** Gradient setup of LC-MS.

Time(min)	Water %(v/v)	Acetonitrile %(v/v)
0.0	5	95
1.0	5	95
7.0	50	50
9.0	50	50
9.1	5	95
13.0	5	95

### Data Availability

The datasets generated during and analyzed during the current study are available from the corresponding author on reasonable request.

## Electronic supplementary material

Supplementary Information
