# Supplementary material for: Metabolic engineering for recombinant major ampullate spidroin 2 (MaSp2) synthesis in Escherichia coli
Source: Sci Rep. 2017 Sep 12;7:11365. doi: 10.1038/s41598-017-11845-2 (PMC5595792; doi:10.1038/s41598-017-11845-2)
Supplement: Supplementary file 1 — Supplementary Information [file 41598_2017_11845_MOESM1_ESM.pdf]

Supplementary Information for Scientific Reports:

Metabolic engineering for recombinant major ampullate spidroin 2 (MaSp2) synthesis in *Escherichia coli*

Hao Cao[1,2], Shafaq Parveen[1], Ding Ding[1], Haijun Xu[1],
Tianwei Tan[1], Luo Liu[1]*

1. Beijing Bioprocess Key Laboratory, Beijing University of Chemical Technology, Beijing, 100029, PR China

2. Institute of Food Science and Technology, Chinese Academy of Agricultural Sciences, Beijing, 100193, PR China

AUTHOR INFORMATION

Corresponding Author

* To whom correspondence should be addressed (Luo Liu).

E-mail: liuluo@mail.buct.edu.cn

Tel: 0086 10 64421335

Fax: 0086 10 64416428

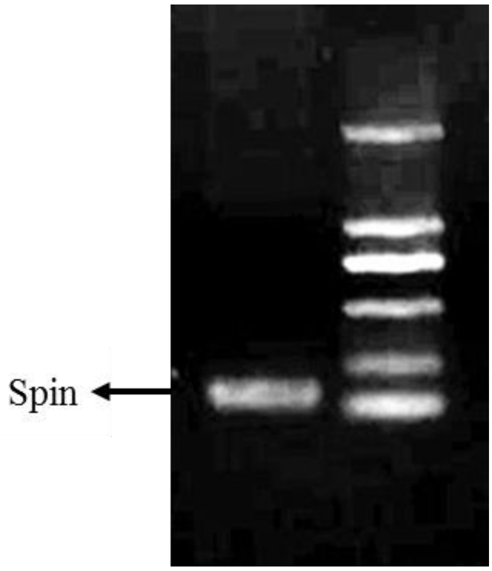
Spin

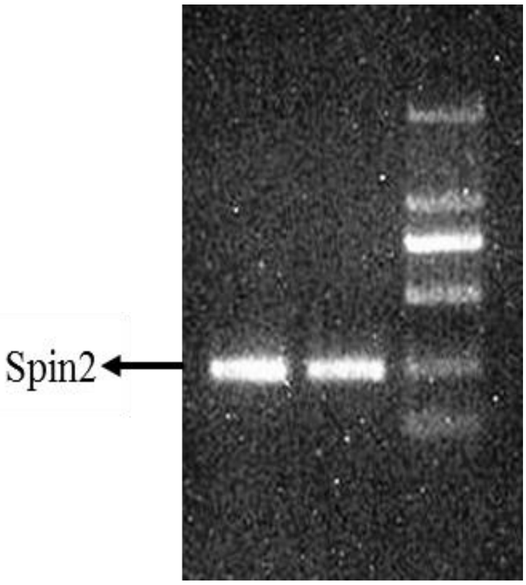
Spin2

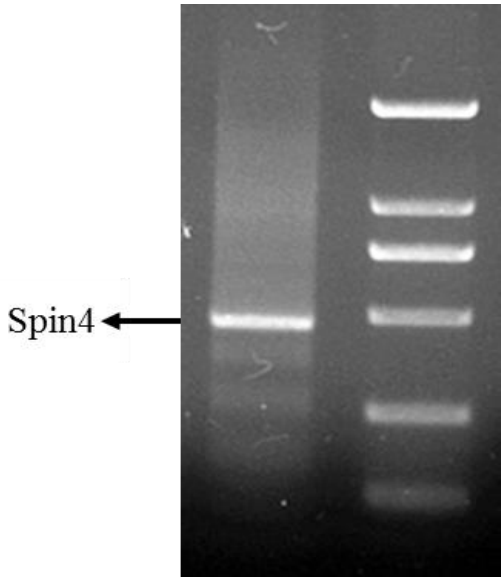
Spin4

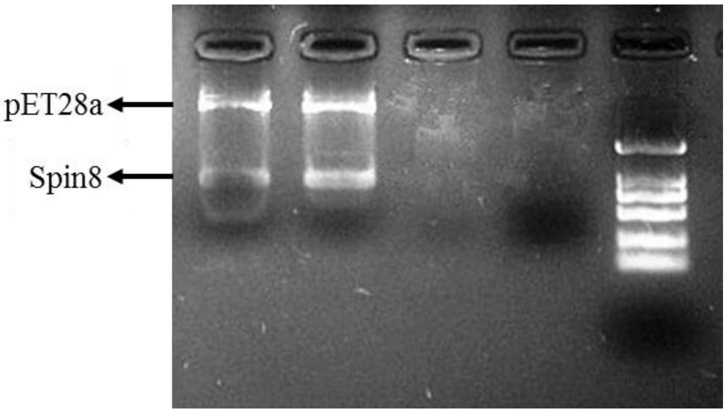
pET28a
Spin8

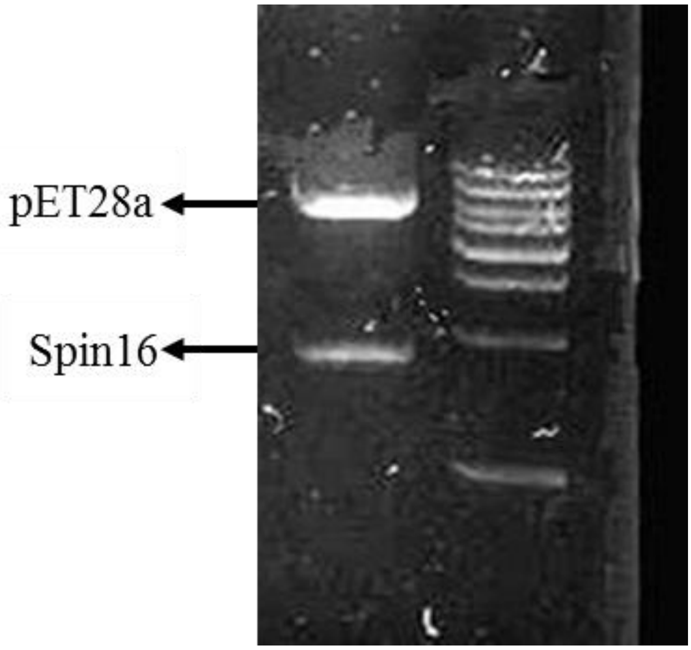
pET28a
Spin16

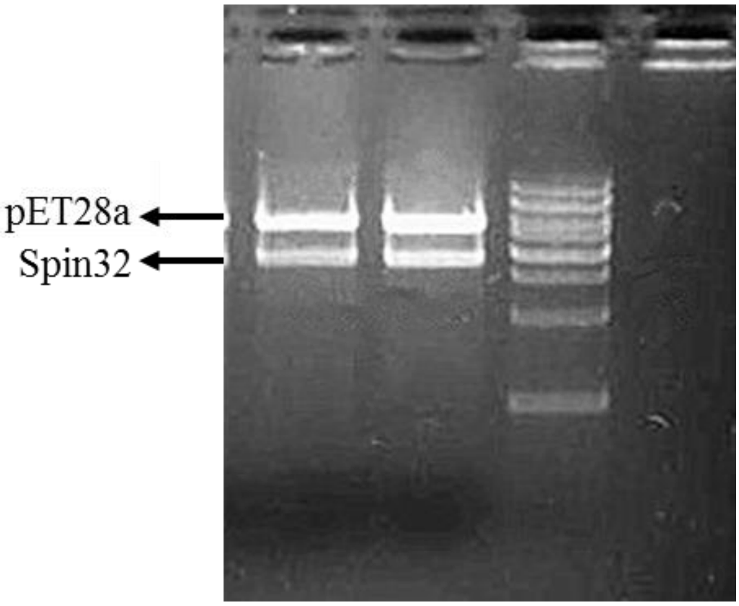
pET28a
Spin32

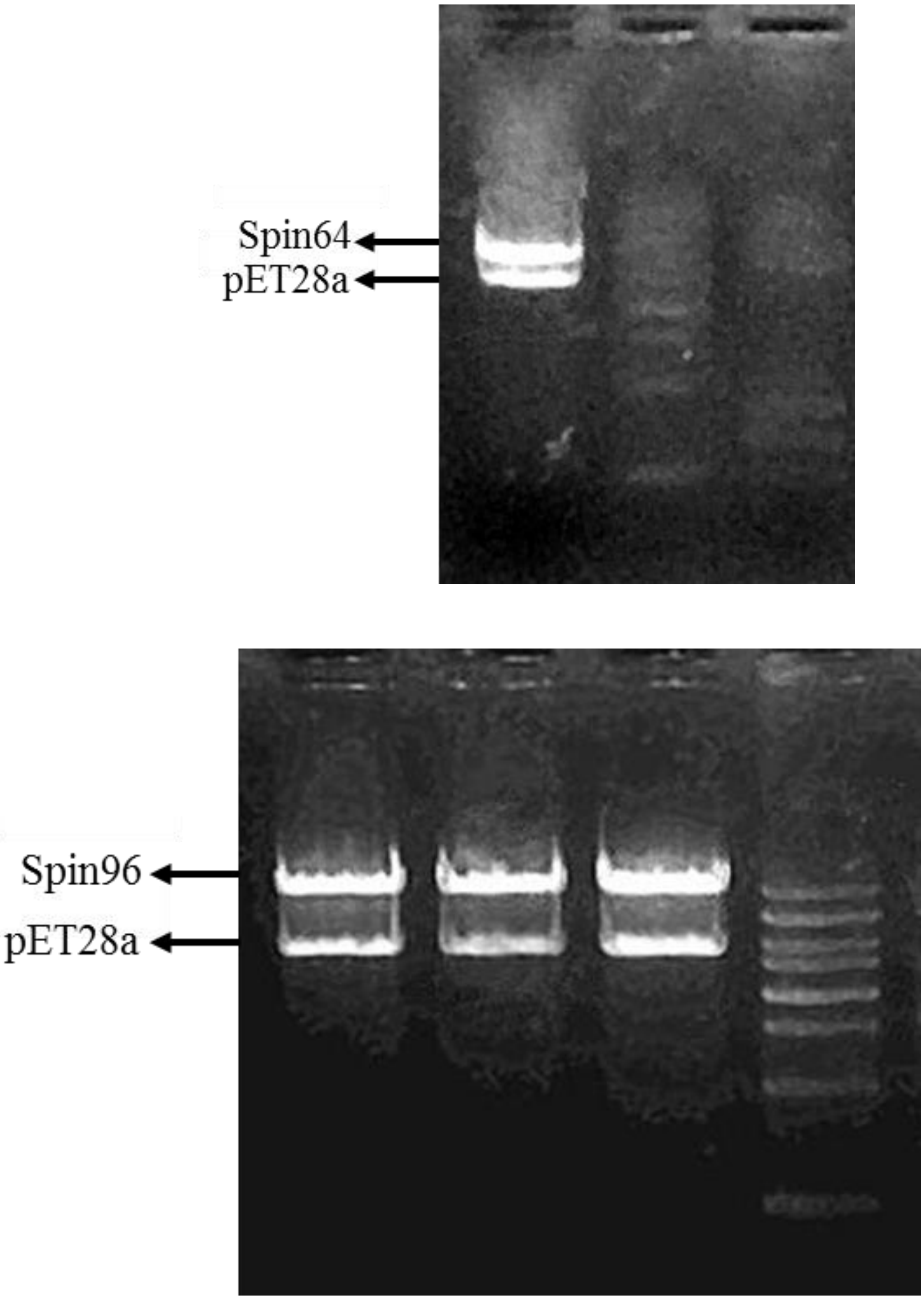

Figured 1. Nucleic acid electrophoresis of the recombinant MaSp2 gene with different tandem spins.
